# LncRNA MALAT1 Affects *Mycoplasma pneumoniae* Pneumonia via NF-κB Regulation

**DOI:** 10.3389/fcell.2020.563693

**Published:** 2020-10-02

**Authors:** Haiyan Gu, Yifan Zhu, Yao Zhou, Tianyu Huang, Siqing Zhang, Deyu Zhao, Feng Liu

**Affiliations:** Department of Respiratory Medicine, Children’s Hospital of Nanjing Medical University, Nanjing, China

**Keywords:** metastasis associated lung adenocarcinoma transcript 1, *Mycoplasma pneumoniae* pneumonia, nuclear factor-κB, inflammation, lncRNA

## Abstract

Our aim was to determine whether the long non-coding RNA (lncRNA) metastasis-associated lung adenocarcinoma transcript 1 (MALAT1) is involved in *Mycoplasma pneumoniae* pneumonia (MPP), and its possible mechanism of action. MALAT1 expression in the bronchoalveolar lavage fluid of 50 hospitalized children with MPP was compared to its expression in 30 children with intrabronchial foreign bodies. MALAT1 expression was higher in children with MPP, accompanied by increased inflammatory mediators interleukin 8 (IL-8) and tumor necrosis factor alpha (TNF-α), compared to the controls. In human airway epithelial cells infected with wild-type *Mycoplasma pneumoniae* (strain M129), MALAT1, IL-8, and TNF-α expression significantly increased, and increased expression of IL-8 and TNF-α could be suppressed by MALAT1 knockdown. Luciferase reporter gene assay and western blot showed that knockdown of MALAT1 reduced nuclear factor-κB (NF-κB) activation. *In vivo*, RNAi packaged with adenovirus (Adv) was nasally transfected into BALB/c mice to silence MALAT1, and an MP-infected mouse pneumonia model was prepared. The results demonstrated that the degree of pulmonary inflammatory injury, vascular permeability, secretion of inflammatory factors, and expression of phosphorylated p65 (pp65) in MP-infected mice were partly reversed after MALAT1 knockdown compared to those in the controls. In conclusion, MALAT1 is involved in the regulation of airway and pulmonary inflammation caused by MP infection via NF-κB regulation.

## Introduction

*Mycoplasma pneumoniae* (MP) is one of the main causes of community-acquired pneumonia in children (Jain et al., 2015). Pneumonia caused by MP often leads to serious complications that affect the quality of life in children (Zhang et al., 2016). In patients with *Mycoplasma pneumoniae* pneumonia (MPP), excessive inflammatory response is a leading cause of pulmonary inflammation and is predictive of poor prognosis. However, the mechanism that leads to the uncontrolled inflammatory response in MPP is still unclear, and further investigation is necessary.

Metastasis-associated lung adenocarcinoma transcript 1 (MALAT1), one of the first lncRNAs associated with human disease to be identified, is of interest because it is widely evolutionarily conserved. MALAT1 was originally identified as a prognostic marker for poor clinical prognosis in patients with early non-small cell lung cancer (NSCLC) (Ji et al., 2003). After that discovery, the function of MALAT1 was studied in various cancers for its role in cancer development and progression (Sun and Ma, 2019). Recent findings suggest that MALAT1 also plays an important regulatory role in inflammatory diseases. For example, MALAT1 was reported to be involved in the regulation of lipopolysaccharide (LPS)-induced acute kidney injury (Ding et al., 2018) and lung injury (Liang et al., 2019). MALAT1 is also an inflammatory regulator in human systemic lupus erythematosus (Yang H. et al., 2017). Downregulation of MALAT1 alleviates saturated fatty acid-induced myocardial inflammatory injury (Jia et al., 2019), while silencing of MALAT1 ameliorated inflammatory injury after lung transplant ischemia-reperfusion (Wei et al., 2019). Therefore, we aimed to determine whether MALAT1 also plays a regulatory role in the inflammatory response in MPP.

Previous reports have shown that MALAT1 regulates the nuclear factor-κB (NF-κB) signaling pathway to modulate the inflammatory response (Lei et al., 2019). MALAT1 affects NF-κB via direct binding or indirect modulation, thus altering the transcription and activation of its downstream inflammatory factors and resulting in varied inflammatory responses (Dai et al., 2018; Ding et al., 2018). Cytokine production in airway epithelial cells can lead to inflammation associated with respiratory diseases. Interleukin-8 (IL-8), a potent chemoattractant and activator of neutrophils, is associated with the initiation and maintenance of inflammation (Pease and Sabroe, 2002). In humans, the airway epithelium is the main source of IL-8, and the pathogenesis of pneumonia is positively correlated with IL-8 expression (Martin et al., 1997). Tumor necrosis factor alpha (TNF-α) is an important bioactive inflammatory mediator that plays a pivotal role in inflammation and immune regulation. High levels of TNF-α can damage capillary endothelial cells, promote microthrombosis, and lead to ischemic necrosis; thus, TNF-α is related to the severity of pneumonia (Salvatore et al., 2007). Therefore, we conducted experiments *in vitro* and *in vivo* to investigate the role of MALAT1 in MPP.

## Materials and Methods

### Study Population

Fifty children who were diagnosed with MPP and underwent bronchoscopy while hospitalized at Children’s Hospital of Nanjing Medical University were included in the study. Thirty children with intrabronchial foreign bodies (FB) were included as the control group. MP infection was diagnosed by polymerase chain reaction (PCR) testing of MP in nasopharyngeal secretions and/or serological testing of MP immunoglobulin M (IgM) by enzyme-linked immunosorbent assay (ELISA). Patients with chronic disease, heart disease, immune deficiency disease, or immunosuppressive drugs were excluded. All enrolled children tested negative for respiratory syncytial virus, influenza virus, parainfluenza virus, pneumovirus, adenovirus, chlamydia trachomatis, and bacterial culture. The study protocol was approved by the Ethics Committee of the Children’s Hospital of Nanjing Medical University (approval number: 201801126-1), and the informed written consent of at least one parent/guardian of each patient was obtained.

### Bronchoalveolar Lavage (BAL)

Guidelines for bronchoscopy and alveolar lavage were reviewed previously (Yang M. et al., 2017; Gao et al., 2018). Children with MPP underwent bronchoscopy and bronchoalveolar lavage fluid (BALF) collection within 1 week after admission. Children with FB were immediately treated with bronchoscopy to remove the foreign bodies, and BALF specimens were collected during reexamination. Irrigation was performed with sterile normal saline (0.3–0.5 mL/kg). Within 1 h after BALF collection, the supernatant was separated, divided, and stored at −20°C. RNA was isolated with TRIzol reagent (Invitrogen, Carlsbad, United States) and stored at −80°C.

### Mycoplasma Culture, Preparation, and Infection

*Mycoplasma pneumoniae* (strain M129) was provided by Professor Chen Z. M. (Children’s Hospital, Zhejiang province), as described in our previous study (Shi et al., 2019). The strain was cultured in a *Mycoplasma* broth, which consists of *Mycoplasma* broth base (Oxoid, Basingstoke, United Kingdom), 0.5% glucose, 0.002% phenol red, and *Mycoplasma* selective supplement G (Oxoid). MP was quantified by counting the number of colony-forming units (CFU) in *Mycoplasma* agar plates.

### Cell Culture and MP Infection

Two human epithelial cell lines, A549 and BEAS-2B (Shanghai Cellular Research Institute, Shanghai, China), were cultured in Dulbecco’s modified Eagle medium (DMEM, Gibco, Grand Island, NY, United States), with 10% fetal bovine serum (FBS, Gibco, Grand Island, NY, United States), in a humidified 5% CO_2_ incubator at 37°C. MP was harvested by centrifugation (10,000 *g* for 20 min), washed, and resuspended in PBS to yield 1 × 10^8^ CFU/mL.A549 and BEAS-2B cells were co-incubated with MP (MOI = 100) for 24 h.

### siRNA and Cell Transfection

SiRNA against MALAT1 and scrambled siRNA were designed and synthesized by RiboBio biotech (RiboBio, Guangzhou, China). Vector transfection was performed using the ribo FECT^TM^ CP transfection kit (RiboBio, Guangzhou, China) in accordance with the manufacturer’s protocol. Sequences of custom siRNA are listed in Table 1.

**TABLE 1:** Sequences for siRNAs.

siNC	5′-UUCUCCGAACGUGUCACGU-3′
siMALAT1-1	5′-CACAGGGAAAGCGAGTGGTTGGTAA-3′
siMALAT1-2	5′-GAG GTGTAAAGGGATTTAT-3′

### Laboratory Animals

Forty male specific-pathogen-free (SPF) BALB/c mice aged 6–8 weeks (body weight: 20–25 g) were purchased from the Laboratory Animals Center of Nanjing Medical University. The mice were housed in conditions with temperatures between 21 and 25°C and relative humidity between 55 and 65%, with free access to food and water. This study was approved by and performed in accordance with the Institutional Animal Care and Use Committee, Nanjing Medical University (IACUC-1904054).

### Adenovirus (Adv)-RNAi Transfection and Subsequent Establishment of Mouse Model of MP Infection

Fluorescent (GFP)-labeled adenovirus packaged with short-hairpin RNA (shRNA) against MALAT1 (Adv-MALAT1*-*RNAi) and scrambled shRNA (Adv-shNC) were synthesized by GeneChem (Shanghai, China). Mice were randomly divided into four groups (*n* = 10 per group): shNC + PBS; sh-MALAT1 + PBS; shNC + MP; and shMALAT1 + MP. To administer the virus, 4% chloral hydrate (0.1 mL/10 g body weight) was given by intraperitoneal to lightly anesthetize the mice, and 30 μL of virus containing 5 × 10^8^ PFU of Adv-shMALAT1-RNAi was inhaled into the lungs through the nose of each mouse. Two doses of this virus were administered to each mouse in 24 h intervals. Mice in the shNC group were treated with control recombinant adenovirus containing scrambled shRNA. After 3 days, mice in the MP group were intranasally administered 10^8^ CFU of M129 in a 50 μL solution, and mice in the PBS group were administered 50 μL of PBS. During administration, their heads were kept tilted at an angle of 30°–45° to ensure fluid flow through the lower respiratory tract. After 4 days, the mice were euthanized, and test samples were collected. Sequences for shRNA are listed in Table 2.

**TABLE 2:** Sequences for shRNAs.

shNC	5′-TTCTCCGAACGTGTCACGT-3′
shMALAT1	5′-GCAGTTTAGAAGAGTCTTTAG-3′

### Biochemical Analysis of Oxidative Stress in Lung Homogenate Samples

After the mice were euthanized, left lung tissue samples were collected, and 10% homogenate was prepared by addition of PBS and centrifugation at 12,000 × *g* at 4°C for 15 min. The supernatant was stored at −80°C for analysis. The concentrations of superoxide dismutase (SOD) and malondialdehyde (MDA) were determined by visible spectrophotometry (Jiancheng Bioengineering Institute, Nanjing, China).

### Collection of Blood and BALF Samples

After euthanasia, 1 mL of blood from each mouse was collected from the eye socket in 1.5 mL Eppendorf tubes. The trachea of each mouse was carefully separated, and 1 mL of normal saline was used to wash and collect the BALF samples three times. Both the blood and BALF samples were centrifuged at 3,000 × *g* at 4°C for 5 min, and the supernatant was stored at −80°C for analysis.

### Hematoxylin-Eosin (HE) Staining

After mice were euthanized using a chloral hydrate injection, specimens of the lower lobe of the right lung were fixed in 4% formaldehyde solution, followed by dehydration, paraffin embedding, sectioning, and HE staining for histopathological analysis.

### Quantitative Real-Time PCR (qRT-PCR)

Total RNA was extracted from cells or mouse lung tissue using TRIzol reagent according to manufacturer’s instructions (Invitrogen, New York, United States). RNA was reverse-transcribed to complementary DNA (cDNA) using PCR Master Mix (Vazyme Biotech, Jiangsu, China). Real-time quantitative PCR was performed using an AceR qPCR SYBR Green Master Mix (Vazyme Biotech, Jiangsu, China). The expression of MALAT1 was normalized to GAPDH and calculated using the 2^–ΔΔCt^ method. The primers used for qRT-PCR are listed in Table 3.

**TABLE 3:** Primers for qRT-PCR.

Human MALAT1-F	5′-CTCCCCACAAGCAACTTCTC-3′
Human MALAT1-R	5′-TTCAACCCACCAAAGACCTC-3′
Human GAPDH-F	5′-GCTCTCTGCTCCTCCTGTTC-3′
Human GAPDH-R	5′-ACGACCAAATCCGTTGACTC-3′
Mouse MALAT1-F	5′-GCGAGCAGGCATTGTGGAGAG-3′
Mouse MALAT1-R	5′-GCCGACCTCAAGAATGTTACCG-3′
Mouse IL-8-F	5′-GGGTGGGGAGTTCGTGTAGA-3′
Mouse IL-8-R	5′-CTACTACACAGGGATCAGGGC-3′
Mouse TNF-α-F	5′-GTGCCAGCCGATGGGTTGTAC-3′
Mouse TNF-α-R	5′-TGACGGCAGAGAGGAGGTTGAC-3′
Mouse GAPDH-F	5′-GGTTGTCTCCTGCGACTTCA-3′
Mouse GAPDH-R	5′-TGGTCCAGGGTTTCTTACTCC-3′

### Enzyme-Linked Immunosorbent Assay (ELISA)

Concentrations of IL-8 and TNF-α in the cell culture supernatant or BALF of children with MPP or FB were detected using the Human IL-8 ELISA Kit (Novus Biologicals, Littleton, United States) and Human TNF-α ELISA Kit (BD Pharmingen, San Diego, United States), respectively. Concentrations of IL-8 and TNF-α in mouse lung homogenate and BALF were detected using the Mouse IL-8 ELISA Kit (Fcmacs Biotech, Nanjing, China) and Mouse TNF-α ELISA Kit (RayBiotech, Atlanta, United States), respectively, according to the manufacturer’s instructions.

### Western Blot

Nuclear protein was extracted from cells and mouse lung tissue using a Nuclear Cytoplasm Extraction Kit (KeyGen Biotech, Nanjing, China). Aliquots of 30 μg of protein lysate were separated using SDS polyacrylamide gel electrophoresis and transferred to PVDF membranes (Millipore, Bedford, MA). After blocking with 5% skim milk at room temperature for 1 h the membrane was incubated overnight with primary antibody at 4°C, and then incubated with secondary antibody at room temperature for 1 h. Finally, ECL reagent (Beyotime, Nanjing, China) was used for chemiluminescent detection. Primary antibodies used for immunoblot analysis included phosphorylated p65 (Cell Signaling Technology, Danvers, United States) and histone H3 (Proteintech, Rosemont, United States).

### Luciferase Reporter Assays

NF-κB transcriptional activity reporter plasmid pNFκB-luc (Beyotime Institute of Biotechnology) and *Renilla* luciferase expression vector pRL-TK (Promega, Madison, United States) were co-transfected into normal or MALAT1-depleted A549 and BEAS-2B cells using Lipofectamine 2000 (Invitrogen, Carlsbad, United States). After 24 h, cells were treated with MP and harvested 24 h later. Luciferase activity was measured using the dual-luciferase reporter assay system (Promega, Madison, United States) in accordance with the manufacturer’s protocol. Relative firefly luciferase activity was normalized to *Renilla* luciferase activity.

### Statistical Analysis

Each experiment was performed in triplicate. All statistical analyses were conducted by SPSS software, version 19.0 (IBM, Armonk, United States). One-way ANOVA, Student’s *t*-test, or Mann-Whitney test were used for statistical comparisons as indicated. *P* < 0.05 was defined as statistically significant.

## Results

### Analysis of MALAT1 Expression in BALF From Children

**FIGURE 1:**
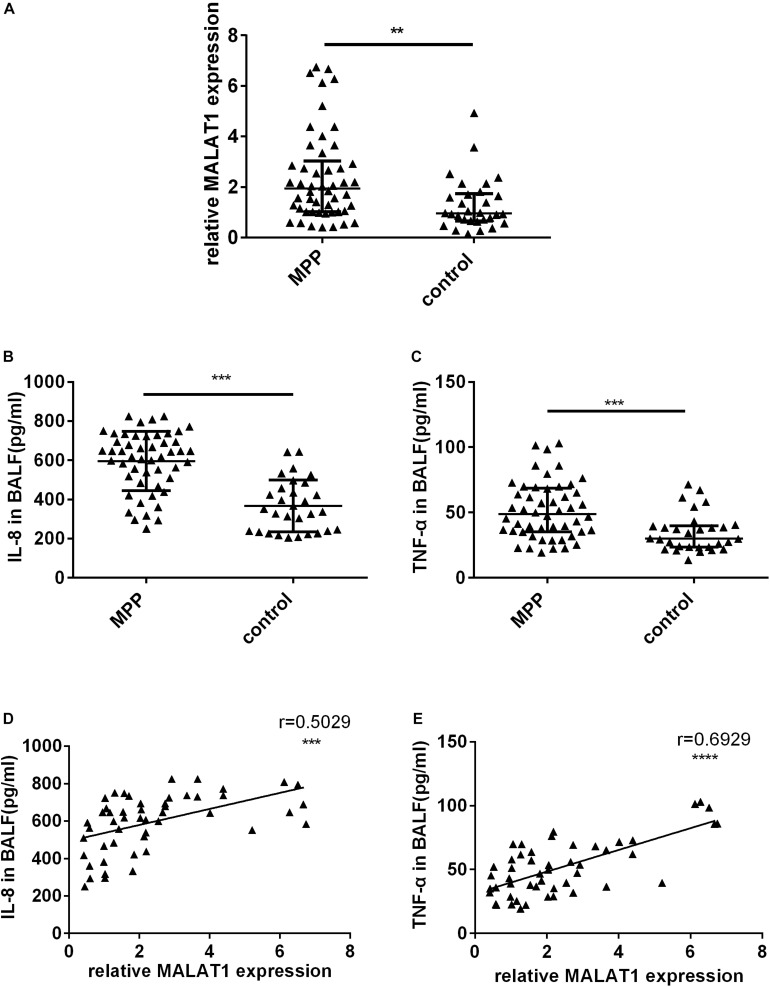
The expression of MALAT1 and inflammatory cytokines in BALF. **(A)** MALAT1 was increased in BALF of 50 children with MPP compared with 30 children with FB. The expression of MALAT1 was measured by qRT-PCR and normalized to GAPDH. Results are shown as median with interquartile range (**P < 0.01 by Mann-Whitney test). **(B,C)** Levels of cytokines (IL-8 and TNF-α) in BALF of children with MPP were much higher than that in BALF of children with FB. Data are shown as mean ± standard deviation **(B)** or median with interquartile range **(C)** (***P < 0.001). **(D,E)** The expression of MALAT1 in BALF of children with MPP was positively correlated with the levels of IL-8 and TNF-α (***P < 0.001, ****P < 0.0001).

First, qRT-PCR was used to detect MALAT1 expression in BALF from children. Expression of MALAT1 in BALF from children with MPP was significantly increased compared to the control group (Figure 1A, *P* < 0.01). In addition, ELISA results suggested that the level of inflammatory factors IL-8 and TNF-α in BALF of the MP group was significantly higher than that of the control group (Figures 1B,C, *P* < 0.001). Further, correlation analysis showed that expression of MALAT1 was positively correlated with expression of IL-8 and TNF-α (Figures 1D,E, *P* < 0.001).

### Knockdown of MALAT1 Inhibited the Increased Secretion of Inflammatory Cytokines by Airway Epithelial Cells After MP Infection

**FIGURE 2:**
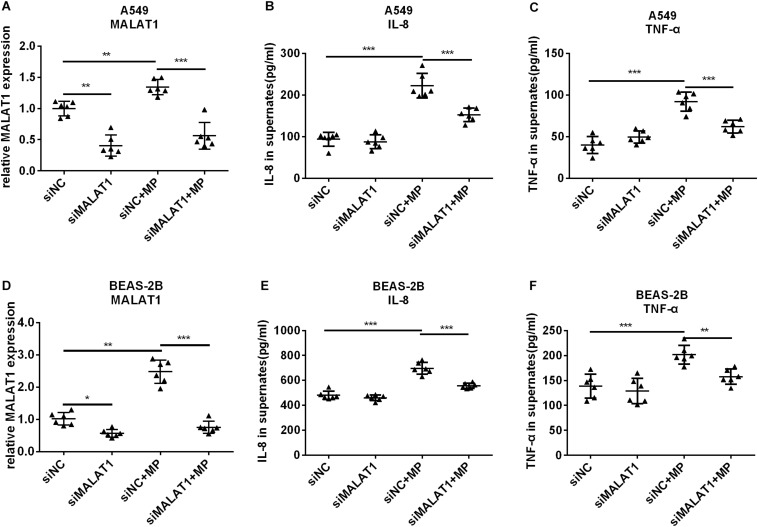
Knockdown of MALAT1 inhibited the secretion of inflammatory cytokines by airway epithelial cells induced by Mycoplasma pneumoniae infection. SiRNA transfection was used to knockdown MALAT1. The expression of MALAT1 was detected by qRT-PCR and normalized to GAPDH. 24 h after MP infection, the expression of MALAT1 in A549 and BEAS-2B cells increased **(A,D)** along with increased levels of IL-8 and TNF-α in cell supernatant **(B,C,E,F)**. The above changes were reversed after MALAT1 was knocked down. Data are shown as mean ± standard deviation (SD) from three experiments (*n* = 6, ***P < 0.001, **P < 0.01, *P < 0.05).

To investigate the role of MALAT1 in MP infection-induced inflammation, siRNA was used to knockdown MALAT1 in A549 and BEAS-2B cells. Then cells were treated with *Mycoplasma pneumoniae* strain M129 for 24 h, and MALAT1 expression was detected using qRT-PCR. As shown in Figures 2A,D, the expression of MALAT1 increased in MP-treated cells, while knockdown of MALAT1 decreased the expression, even after MP infection (all *P* < 0.05). In addition, the levels of inflammatory factors in the cell supernatant were detected using ELISA. The results demonstrated that levels of IL-8 and TNF-α secreted by A549 and BEAS-2B cells were increased after MP infection compared to the control group, while the inflammatory cytokines levels decreased in MALAT1-depleted epithelial cells compared to those of the non-knockdown group after MP infection (Figures 2B,C,E,F, all *P* < 0.05).

### Knockdown of MALAT1 Inhibited NF-κB Activation Induced by MP Infection in Airway Epithelial Cells

**FIGURE 3:**
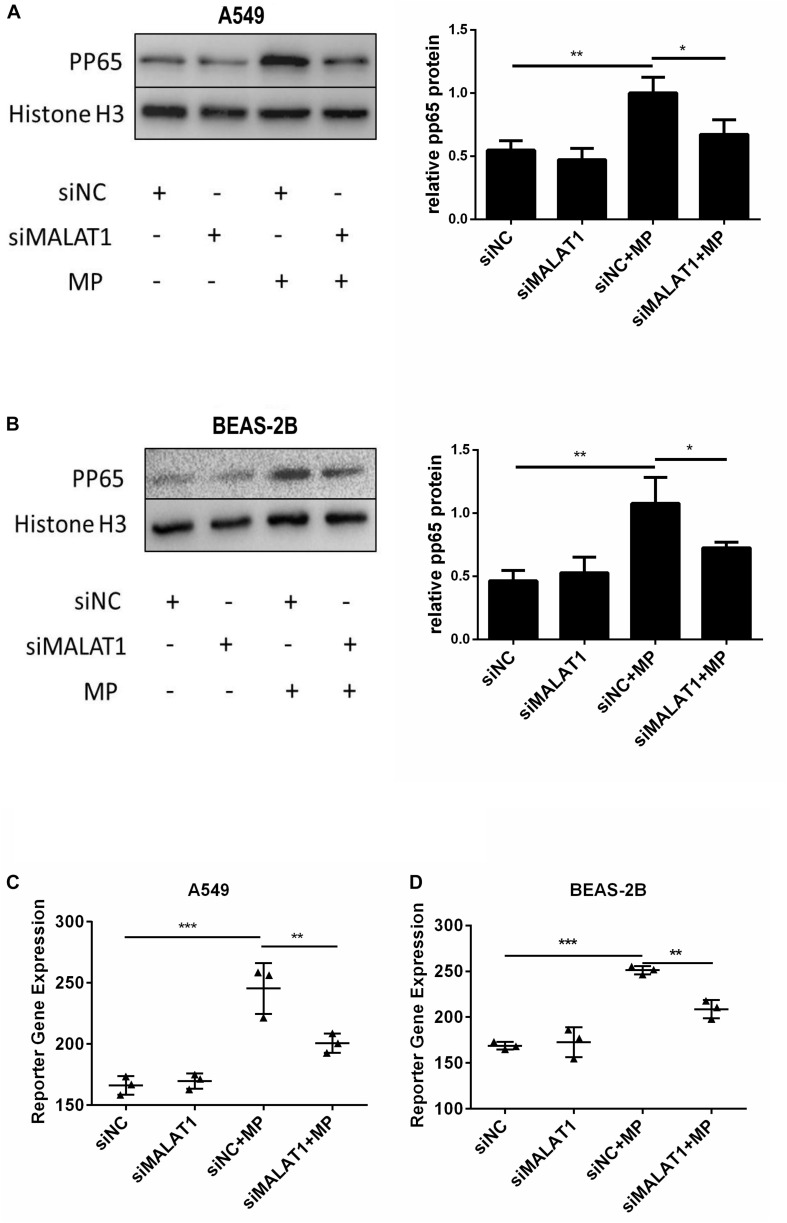
Knockdown of MALAT1 inhibited NF-κB activation of airway epithelial cells induced by MP infection. The expression of phosphorylated p65 protein in A549 **(A)** and BEAS-2B **(B)** cells was detected by western blot. Compared with the normal group, the expression of phosphorylated p65 decreased after MP infection in the knockdown group. The activity of NF-κB was measured by the dual- luciferase reporter gene assay. Compared with the control group, the NF-κB activity of the MALAT1 silent group decreased after MP infection **(C,D)**. Data are shown as mean ± standard deviation (SD) from three experiments (*n* = 3, ***P < 0.001, **P < 0.01, *P < 0.05).

When NF-κB is activated, the subunit of NF-κB, p65, is phosphorylated and translocated to the nucleus. Therefore, we detected pp65 in the nucleus of A549 and BEAS-2B cells by western blot, and the results showed that MALAT1 knockdown reduced pp65 expression in the nucleus of MP infected cells (Figures 3A,B, *P* < 0.05). This is in accordance with the results of the dual luciferase reporter assay (Figures 3C,D, *P* < 0.01). These results suggest that MALAT1 knockdown inhibited NF-κB activation induced by MP infection in airway epithelial cells.

### MALAT1 Knockdown Reduced Histopathological Damage Caused by MP Infection in BALB/c Mice

**FIGURE 4:**
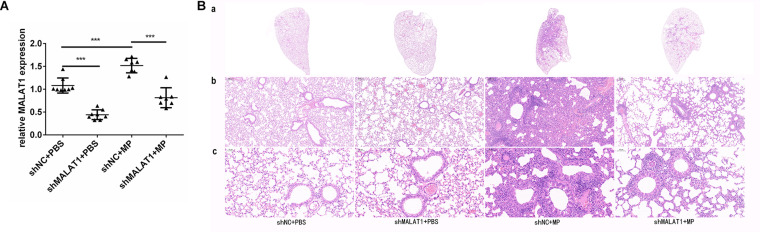
MALAT1 knockdown reduced histopathological damage in BALB/c mice caused by MP infection. Lung tissue samples were collected from each group of mice, then the expression of MALAT1 was detected by qRT-PCR. **(A)** The expression of MALAT1 in the lung tissues of mice was up-regulated in shNC + MP group, while it was decreased in shMALAT1 + PBS group, compared to the shNC + PBS group. In addition, the expression of MALAT1 was inhibited in shMALAT1 + MP group compared with the shNC + MP group. Data are shown as mean ± standard deviation (SD) from three experiments (*n* = 8, ***P < 0.001). **(B)** Histopathological changes were evaluated by HE staining of lung-tissue samples. The shNC + MP group showed significant histological changes, including cell infiltration, alveolar wall thickening, significantly increased secretion, and even structural collapse, while the above changes were reversed in shMALAT1 + MP group. (a) Representative scans (original magnification × 5) of HE stained FFPE sections of left lungs; (b) digital zoom of the lung mid region; (c) representative images (original magnification × 200) for histopathology changes (*n* = 6).

Lung tissue samples were collected from each group of mice, and MALAT1 expression was detected using qRT-PCR. The expression of MALAT1 was upregulated in the shNC + MP group and downregulated in the shMALAT1 + PBS group, compared to the shNC + PBS group. In addition, the expression of MALAT1 was decreased in the shMALAT1 + MP group compared to the shNC + MP group (Figure 4A, *P* < 0.001). As shown in Figure 4B, histopathological changes were evaluated by HE staining of the lung tissue samples. The shNC + MP group showed significant histological changes, including cell infiltration, alveolar wall thickening, significantly increased secretion, and even structural collapse. These changes were reversed upon MALAT1 knockdown in the infected group.

### MALAT1 Knockdown Reduced Lung Injury Caused by MP Infection in BALB/c Mice

**FIGURE 5:**
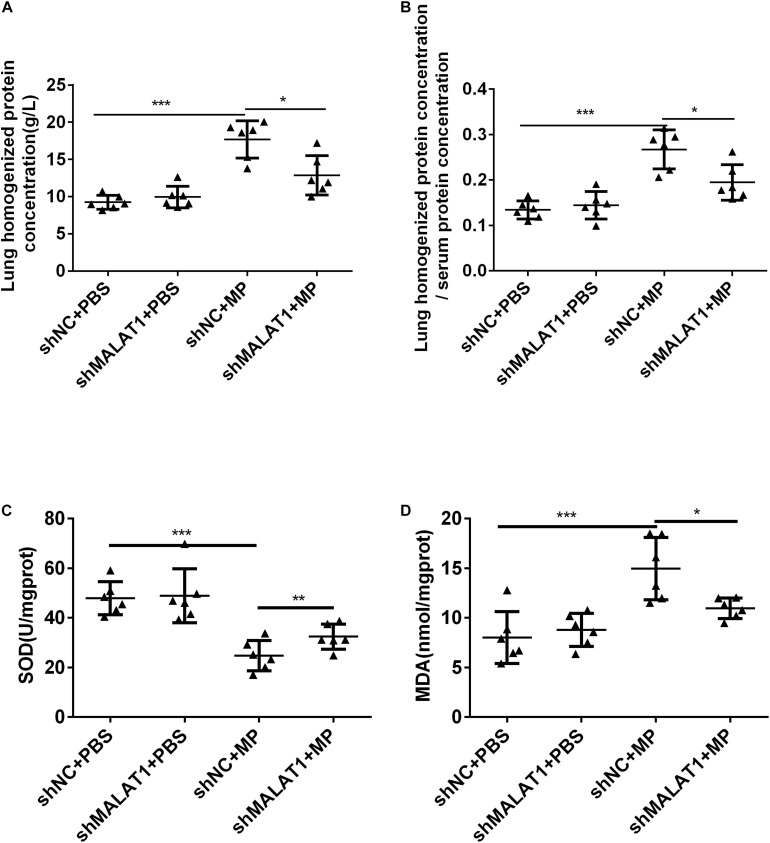
MALAT1 knockdown reduced lung injury in BALB/c mice caused by MP infection. **(A,B)** The shNC + MP group mice exhibited increases in protein concentration in lung homogenate and ratio of protein concentration in lung homogenate and serum compared to the shNC+PBS group. The two indexes were decreased upon MALAT1 knocked down in MP infected group. **(C,D)** Compared with shNC + MP group, SOD level in lung homogenate of mice in the shMALAT1 + MP group was significantly increased, while MDA level was decreased. Data are shown as mean ± standard deviation (SD) from three experiments (*n* = 6, ***P < 0.001,*P < 0.05).

After MP infection, the shNC + MP group exhibited increases in protein concentration in lung homogenate and ratio of protein concentration in lung homogenate and serum. In terms of these two indexes, the shNC + MP group exhibited a greater increase than the shMALAT1 + MP group (Figures 5A,B, *P* < 0.05). The level of SOD activity indirectly reflects the body’s ability to remove oxygen free radicals, and the level of MDA indirectly reflects the severity of free radical attack on cells. The results showed that compared with the shNC + MP group, the SOD activity of mice in the shMALAT1 + MP group significantly increased, while the MDA concentration decreased (Figures 5C,D, *P* < 0.05).

### MALAT1 Knockdown Reduced the Expression of Inflammatory Cytokines in Mice With MP Infection

**FIGURE 6:**
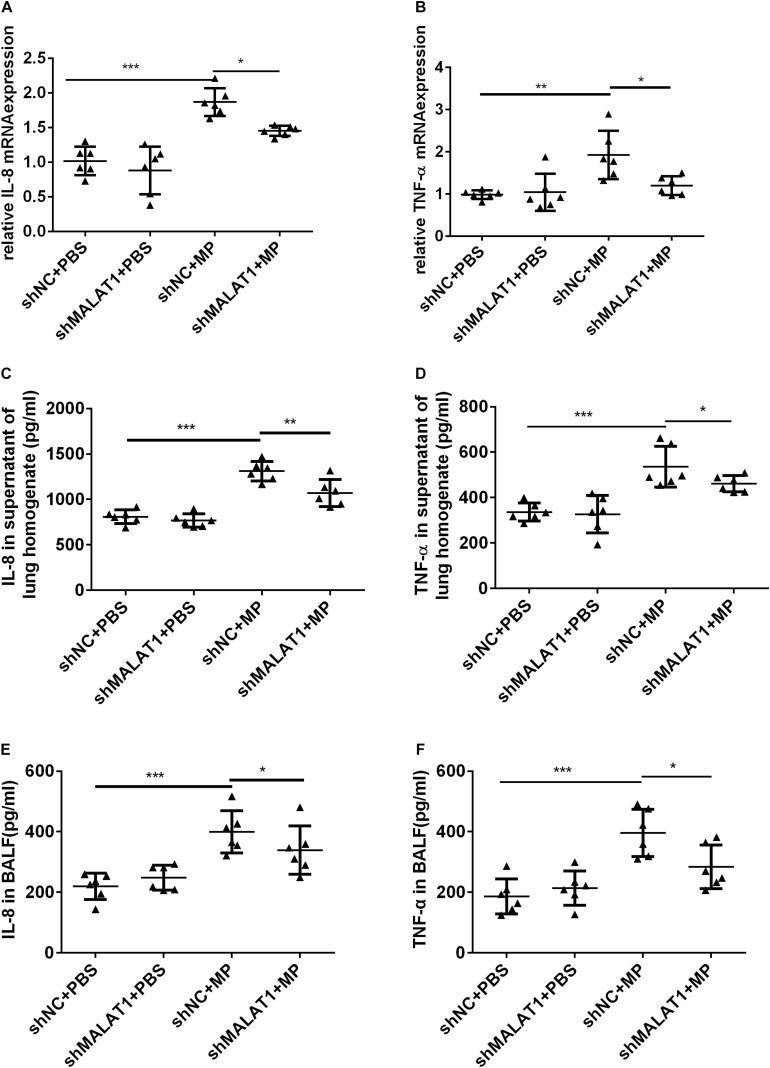
MALAT1 knockdown reduced the expression of inflammatory cytokines in mice with MP infection. The mRNA expressions of IL-8 and TNF-α in lung tissues were detected by qRT-PCR **(A,B)**, and the protein level of IL-8 and TNF-α in mice lung homogenate **(C,D)** and BALF **(E,F)** were detected by ELISA. Data are shown as mean ± standard deviation (SD) from three experiments (*n* = 6, ***P < 0.001, **P < 0.01, *P < 0.05).

The mRNA expression of IL-8 and TNF-α in mouse lung tissue was detected using qRT-PCR, and the protein expression of IL-8 and TNF-α in mouse lung homogenate and BALF was detected using ELISA. The results showed that both the mRNA and protein expression of IL-8 and TNF-α in the shNC + MP group significantly increased compared to the shNC + PBS group, and knockdown of MALAT1 reversed the increase (Figure 6, all *P* < 0.05).

### NF-κB Is Involved in MALAT1-Mediated Regulation of the Inflammatory Response Induced by MP Infection

**FIGURE 7:**
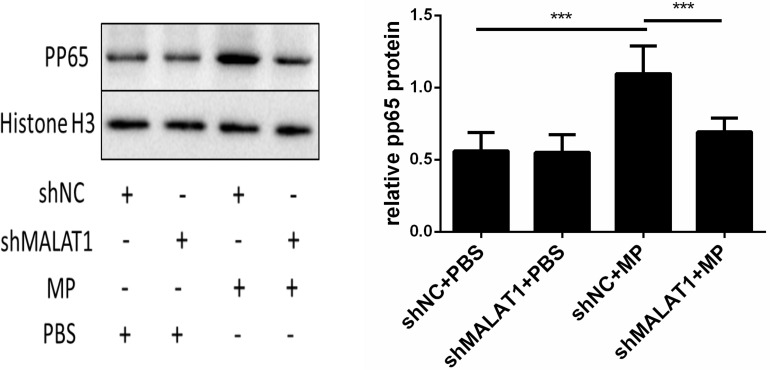
NF-κB is involved in the role of MALAT1 in regulating the inflammatory response induced by MP infection. The expression of phosphorylated p65 in lung tissues of each group was detected by western blot. Densitometry results of the western-blot are shown in the bar graph. The expression of phosphorylated p65 in the shNC + MP group was significantly increased, comparing with the shNC + PBS group, however, the above change was reversed after MALAT1 knocking down. Data are shown as mean ± standard deviation (SD) from three experiments (*n* = 6, ***P < 0.001).

Using western blot, we detected phosphorylated p65 expression in the lung tissue of each group. As shown in Figure 7, expression of phosphorylated p65 in the shNC + MP group was significantly increased compared to the shNC + PBS group; however, the above change was reversed after MALAT1 knockdown. These results suggested that the NF-κB signaling pathway is involved in MALAT1-mediated regulation of the inflammatory response induced by MP infection.

## Discussion

MP infection may cause severe local inflammation, leading to cytotoxicity and tissue damage. For this reason, patients with MPP are prone to atelectasis and necrotizing lesions and often require fiberoptic bronchoscopy. According to previous studies, the local inflammatory response is initiated by the release of pro-inflammatory cytokines from epithelial cells (Yang et al., 2002). Among the induced cytokines, IL-8 and TNF-α play pivotal roles in the inflammatory response of MPP. Our data showed that the levels of IL-8 and TNF-α were significantly elevated in BALF from children with MPP, supernatants of MP-infected epithelial cells, and lung tissues and BALF of mice infected with MP compared to the non-infected groups. These results confirmed the role of IL-8 and TNF-α in airway inflammation caused by MPP.

MALAT1 has been reported to be involved in multiple diseases. Recently, researchers have focused on its role in inflammatory diseases. Based on the findings in this study that MALAT1 was highly expressed and expression was positively correlated with inflammatory factors IL-8 and TNF−α in BALF from children with MPP, we performed *in vitro* and *in vivo* experiments to determine the role of this lncRNA in MP-induced inflammation. Our *in vitro* results showed that MALAT1 knockdown inhibited the secretion of inflammatory factors from epithelial cells after MP stimulation. *In vivo*, after MP infection, the expression of IL-8 and TNF-α in lung homogenate and alveolar lavage fluid increased. SOD activity in lung homogenate decreased, while MDA concentration increased, indicating the existence of anti-oxidation imbalance. The ratio of lung homogenate protein concentration to serum total protein concentration was significantly increased, indicating the presence of pulmonary permeability damage, which is consistent with the previous study (Shi et al., 2019). However, MALAT1 knockdown in BALB/c mice alleviated lung inflammation and lung injury induced by MP infection. These results demonstrated for the first time the regulatory role of MALAT1 in MP-induced inflammation.

Previous studies have shown that the regulatory role of MALAT1 in inflammation is complex. For instance, a recent study reported that MALAT1 knockdown markedly reduced lung injury induced by sepsis (Lin et al., 2019). Furthermore, knockdown of MALAT1 was reported to suppress inflammatory response in LPS-induced acute lung injury and kidney injury (Dai et al., 2018; Ding et al., 2018; Cui et al., 2019; Liang et al., 2019). Knockdown of *Malat1* in mice alleviated inflammatory injury after cerebral ischemia, and overexpression of *Malat1* aggravated ischemic brain inflammation (Cao et al., 2019). In addition, MALAT1 has been reported to promote inflammation in septic heart damage (Chen et al., 2018), systemic lupus erythematosus (Yang H. et al., 2017), and hyperglycemia-induced inflammatory process (Puthanveetil et al., 2015). The above findings together with the results from the current study suggest that MALAT1 plays a pro-inflammatory role in many diseases. However, other studies demonstrated that lncRNA MALAT1 performs a protective function against inflammation in several diseases (Zhao et al., 2016; Cremer et al., 2019; Sun et al., 2019; Zhu and Men, 2019). These inconsistencies may be due to the complex mechanisms of MALAT1 regulation in different diseases.

With respect to the mechanism of MALAT1, our study indicated that the pro-inflammatory function of MALAT1 at least in part depends on NF-κB. NF-κB is involved in the inflammatory response of MPP. The lipoprotein components of MP recognize toll-like receptors and activate NF-κB (Shimizu et al., 2007, 2008). In our present study, we detected NF-κB activation in MP-infected epithelial cells and mouse lung tissue and confirmed that MP infection activates NF-κB. According to previous studies, MALAT1 can regulate inflammation by modulating the activation of NF-κB. MALAT1 can bind directly to the subunit of NF-κB (Zhao et al., 2016; Zhu and Men, 2019) or act as competing endogenous RNA (ceRNA) and compete with miRNA functions toward NF-κB (Ding et al., 2018; Cao et al., 2019; Jia et al., 2019; Li et al., 2019; Liang et al., 2019). Our data suggested that MALAT1 knockdown inhibits phosphorylation of p65 caused by MP infection in airway epithelial cells and lung tissue of mice. Therefore, the regulatory role of MALAT1 in inflammation of MPP is associated with NF-κB activation. Although the precise mechanism remains unclear, acting as a certain ceRNA might be the way by which MALAT1 facilitate p65 phosphorylation in MP infection. Further study is needed to reveal the exact regulatory mechanism.

## Conclusion

In conclusion, our present study demonstrates that lncRNA MALAT1 plays a key regulatory role in MP-induced inflammation. The regulatory function of MALAT1 likely proceeds through NF-κB signaling. By performing *in vivo* experiments, we found that downregulation of MALAT1 reduces pulmonary inflammation caused by MP infection. Our results suggest that MALAT1 may be a new therapeutic target for MPP.

## Data Availability Statement

All datasets generated for this study are included in the article.

## Ethics Statement

The studies involving human participants were reviewed and approved by the Ethics Committee of the Children’s Hospital of Nanjing Medical University. Written informed consent to participate in this study was provided by the participants’ legal guardian/next of kin. The animal study was reviewed and approved by the Institutional Animal Care and Use Committee, Nanjing Medical University.

## Author Contributions

HG performed the experiments and statistical analysis, contributed to the interpretation of data, made the figures and tables, and drafted the manuscript. YiZ participated in study design, collected and interpreted the clinical information, and determined the clinical status for each children involved in the study. YaZ, TH, and SZ participated in animal experiments and data interpretation. DZ and FL designed the study, analyzed the data, and revised the manuscript. All authors contributed to the article and approved the submitted version.

## Conflict of Interest

The authors declare that the research was conducted in the absence of any commercial or financial relationships that could be construed as a potential conflict of interest.
